# Evaluation of the trade-off between variety, processing, and low-GI claim in ready-to-eat rice

**DOI:** 10.1177/10820132241272768

**Published:** 2024-08-11

**Authors:** Diva Cabral, Susana C Fonseca, Célia Rocha, Ana P Moura, Jorge C Oliveira, Luís M Cunha

**Affiliations:** 1 GreenUPorto/Inov4Agro, DGAOT, 131674 Faculty of Sciences, University of Porto, Vila do Conde, Portugal; 2 SenseTest – Sociedade de Estudos de Análise Sensorial a Produtos Alimentares, Vila Nova de Gaia, Portugal; 3 GreenUPorto/Inov4Agro, DCeT, 467191 Universidade Aberta, Porto, Portugal; 4 School of Engineering and Architecture, 155281 University College Cork, Cork, Ireland

**Keywords:** Rice, ready-to-eat, low-GI claim, bran, naturalness, well-being, convenience

## Abstract

An increasing number of consumers demand healthier, more convenient, and sustainable food products, including rice, a staple worldwide. Food manufacturers have responded to this trend by considering food’s intrinsic and extrinsic aspects. This study evaluated the importance of variety, processing, and claims on willingness to try ready-to-eat rice (RTE-rice). It also analyses the influence of consumer attitudes on the importance of attributes and willingness to try. The results showed that processing significantly influenced willingness to try RTE-rice, revealing consumers’ greater preference for whole grain than milled rice with added bran. Claims had the least relevant importance. However, low glycaemic index had a positive impact, indicating its potential to influence consumer purchasing attitudes and promote healthier rice consumption. Additionally, three groups were created based on attitudinal factors. Naturalness-oriented and convenience-oriented groups were more likely to try RTE-rice. However, the reasons that motivate them may be different; this latter could be the ease of the service offered, while for the group focused on naturalness, they may have perceived through the ingredients and claimed that the product, despite being convenient, can bring benefits, thus perceiving them as natural.

## INTRODUCTION

Rice is one of the most important staple foods in the world (Worldatlas, 2019), and is consumed mostly in milled form (white rice) which is richer in starch and poorer in other nutrients such as protein, fibre, vitamins, mineral salts, and phytochemical components than its whole grain counterpart (Mir et al., 2020). Rice provides approximately 25% of the world's daily calorie needs and has been considered as a solution to eradicate hunger in resource-limited countries (Yadav and Kumar, 2018). It is an excellent vector for micronutrient fortification (Dipti et al., 2012) and has been vital in fighting malnutrition. In addition to its role in food security, rice is considered one of the most strategic commodities because of its low price, high yield, and support to the subsistence of many families in rural areas of developing countries (Yadav and Kumar, 2018). However, the most common form of consumption, white rice, has been linked to various chronic diseases, such as type 2 diabetes, obesity, and other metabolic syndromes, owing to its high glycaemic index (Bhavadharini et al., 2020; Golozar et al., 2017; Liu et al., 2022; Saneei et al., 2017; Seah et al., 2019; Yu et al., 2022).

Recent years have seen nutritional challenges due to excessive consumption fuelled by sociodemographic and lifestyle changes (Dipti et al., 2012). Changes in the human social environment, disposable income, and food supply and demand have altered eating patterns, whereby consumers turn to more convenient products to cope with a faster-paced lifestyle (Buckley et al., 2007). The main challenges to society's eating patterns are deeply intertwined with the loss of skills in preparing meals from scratch. This skill is influenced by cultural, social, familial, professional, and educational situations (Lavelle et al., 2016). Busier lifestyles, long working hours, and multiple commitments leave less time to cook from scratch, so there is an appreciation for ready-made or quickly prepared food. These changes reduce the need for culinary skills and decrease their transmission of culinary skills. Thus, the population has been losing the necessary skills to prepare meals from scratch (Worsley et al., 2015), leaving aside healthy food (Reipurth et al., 2019).

In addition to the importance of health and convenience in food choices, there has been a growing awareness of food waste and its environmental impacts (Alsaffar, 2016; Bisoffi et al., 2021; FAO, 2022; Griffen, 2020), including the origin of raw materials (Gonçalves and Maximo, 2023; Lucarini et al., 2020). Incorporating by-products as ingredients and using local products are strategies to achieve this goal (Jurgilevich et al., 2016).

To overcome these issues, industries and researchers are focusing on healthier, more convenient, and sustainable diets (Bisoffi et al., 2021; Hassoun et al., 2022). The combination of convenience and health has been considered incompatible for many years because of the negative perceptions of the effects of processed foods on health (Lavelle et al., 2016; Peura-Kapanen et al., 2017; Rogus, 2018). The EAT-Lancet Commission has reinforced that convenient and healthy products must be available for staple foods such as rice, to help with everyday life without harming consumer health (Willett et al., 2019). To achieve more sustainable products, food manufacturers must consider the origin of raw materials (Gonçalves and Maximo, 2023; Lucarini et al., 2020), the incorporation of by-products as ingredients, and the use of local products (Jurgilevich et al., 2016).

In this study, we evaluated the willingness to try (WTT) ready-to-eat rice (RTE-rice) products, considering variety, processing, and label claims as attributes for designing healthy and convenient rice products. In addition, we explored the relationship between the importance of attributes and consumer attitudes.

Brown rice and milled rice supplemented with bran, both rich in dietary fibre, were used as the processing attributes. Fibres play an essential role in reducing the glycaemic response of rice (Augustin et al., 2015; Chiu et al., 2011; Nakamura et al., 2022). Brown rice is obtained by dehusking while maintaining the bran, while milled rice loses bran, germ, and nutritional value due to the milling process. Brown rice is less popular because of its texture, colour, flavour, and longer cooking time (Gondal et al., 2021; Zhou et al., 2019), however, it is a healthier alternative to milled rice.

Rice bran, a by-product that accounts for 5–10% of paddy rice weight, is rich in nutrients such as protein, fat, dietary fibre, minerals (potassium, calcium, magnesium, and phosphorus), and bioactive components (tocopherols, tocotrienols, and γ-oryzanol) (Castanho et al., 2019; Saji et al., 2019; Wang et al., 2023). These compounds possess antioxidant, anti-inflammatory, hypocholesterolaemia, antidiabetic, and anticancer properties (Sohail et al., 2017; Tan et al., 2023). Due to its nutraceutical properties, rice bran has potential applications in the pharmaceutical and food industries, including the development of cooking oils, food colouring, edible coatings, and bakery products (Bodie et al., 2019; Tan et al., 2023; Yadav et al., 2021).

Informative labelling, such as the health benefits of food products, can significantly influence consumers’ food choices (Ballco et al., 2020; Hallez et al., 2023; Jindahra and Phumpradab, 2023). Since 1998, WHO and FAO have recommended using glycaemic index (GI) as a valuable indicator for choosing a healthy diet. They advise consumers to opt for foods rich in non-starch polysaccharides and low in GI (FAO/WHO, 1998). High-GI diets have been linked to an increased risk of developing chronic non-communicable diseases, and some countries have addressed this issue in their health policies (Barclay et al., 2021).

## MATERIAL AND METHODS

### Participants

Sampling was selected based on the age and sex quota, with the following inclusion criteria: (i) being Portuguese, (ii) consuming rice at least three times per week, and (iii) being responsible or sharing responsibility for grocery shopping and preparing meals. Participants were recruited from the sensory evaluation company, Sense Test's consumer database (Vila Nova de Gaia, Portugal). They were mainly residents of the Porto metropolitan area in northern Portugal.

All participants provided informed consent before participating, which was approved by the Ethical Committee of the *Faculdade de Ciências da Universidade do Porto* (reference number 50/2023). This work was undertaken with the support of the Sense Test Company, ensuring the protection and confidentiality of data through the authorisation 2063/2009 of the National Data Protection Commission. Internal conduct, following the General Data Protection Regulation standards and implementing informed consent was accomplished. All the participants received a small amount of financial compensation for their participation.

### Experimental design

A structured questionnaire was administered using the Lime Survey software, which comprised five sections: conjoint task, perception of well-being related to rice choice, the importance of naturalness and convenience in rice choice, and sociodemographic characteristics. The questionnaire was completed in person using a computer provided by the recruitment company. This study identified eight different combinations of RTE-rice, based on three attributes: variety, processing, and claims. These attributes and their levels were determined using inputs from previous studies on rice consumption, perception of brown rice, and expectations of low glycaemic index (low-GI) rice products (Cabral et al., 2024a).

Basmati and *Carolino* rice varieties comprise the levels of the variety attribute. Basmati rice, which has a low-GI (Atkinson et al., 2021), has gained popularity among Portuguese consumers, while *Carolino* rice, the local rice, has experienced a decline in consumption (Cabral et al., 2024a; Castanho et al., 2023). Brown rice and milled rice supplemented with bran, both high in dietary fibre, were used for processing. Claims play a crucial role in informing consumers about the intrinsic properties of food. Two levels of claims were utilised: the source of the fibre and the low-GI.

**Figure 1.**
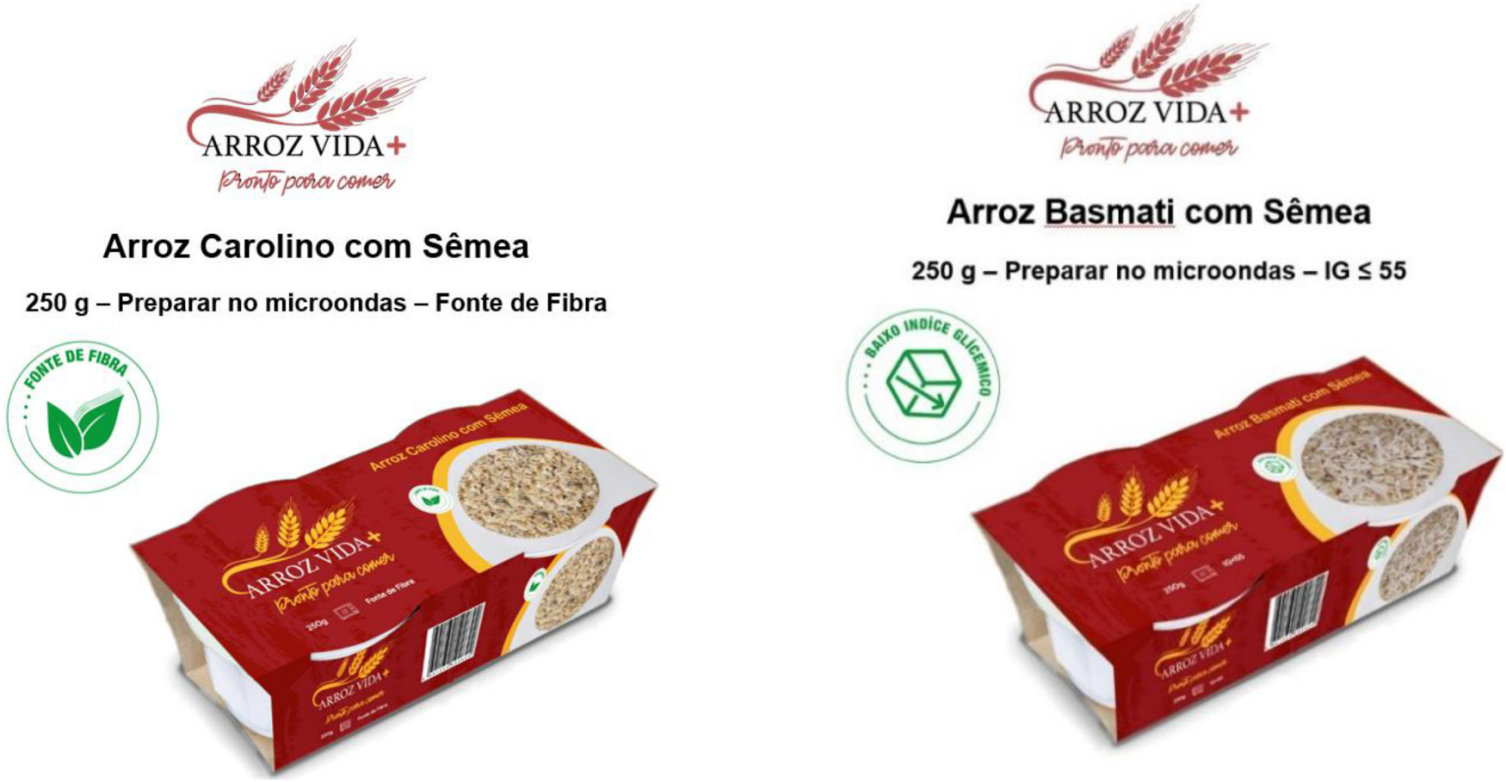
Examples of the cards (in Portuguese) used in the conjoint analysis exercise, corresponding to profile #4 (left) and profile #1 (right), as coded in Table 1.

All attributes were integrated as text and symbols in mock-up package images. Eight digital cards with packaging images were designed using Adobe Photoshop^®^ software 2022 v. 24.7.1 (Figure 1).

**Table 1.** Selected profiles created by combining different levels of the attributes: variety, processing, and claims.

	Variety		Processing		Claim	
Profile #	*Carolino*	Basmati	Brown	Milled + bran	Low-GI	Source of fibre
1	No	Yes	No	Yes	Yes	No
2	Yes	No	No	Yes	Yes	No
3	No	Yes	No	Yes	No	Yes
4	Yes	No	No	Yes	No	Yes
5	No	Yes	Yes	No	Yes	No
6	Yes	No	Yes	No	Yes	No
7	No	Yes	Yes	No	No	Yes
8	Yes	No	Yes	No	No	Yes

**Figure 2.**
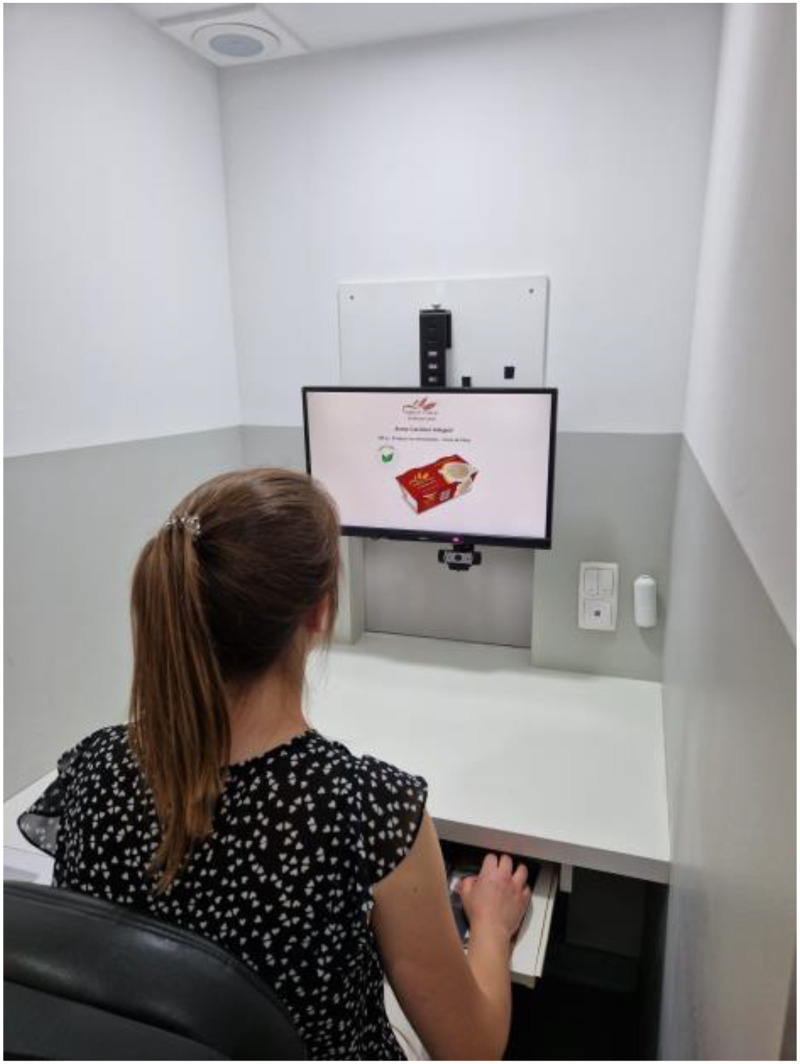
Photo illustration of the setup used for the conjoint task.

Each card was coded with a three-digit random number presented to the participants, as depicted in Figure 2, and a sequential monadic presentation was implemented in balanced order (Macfie et al., 1989). The experiment commenced with a welcoming introduction and brief overview of the symbols and information displayed in the packaging of RTE-rice to ensure a uniform understanding of the experimental attributes. Each aspect of the packaging was thoroughly explained to the participants. They were then asked to rate their WTT the product on a 9-point scale ranging from ‘extremely unwilling’ to ‘extremely willing’.

### Attitudinal evaluation

Previous studies have shown that in addition to healthiness, convenience and naturalness are values consumers consider essential in a rice product with a healthier profile and label information (Cabral et al., 2024a). Therefore, the importance levels of these dimensions were measured using established measures. The importance of naturalness in choosing more often consumed rice was measured using a New Naturalness Scale (NNS) consisting of nine items (Michel and Siegrist, 2019). Seven items were selected from convenience food lifestyle (CFL) to evaluate consumer convenience orientation (Buckley et al., 2007). Twelve items of the well-being scale developed by Ares et al. (2016) were used to assess consumer perceptions of well-being with the rice they frequently choose. These items were selected based on the results of a previous study that evaluated the perception of well-being of different RTE-rice varieties available in the Portuguese market (Cabral et al., 2024b). This study assessed the perception of well-being of five different RTE-rice varieties, based on *Carolino* and Basmati varieties: whole, milled, or mixed with seeds and other cereals. Only the items perceived as significantly different among the different RTE-rice samples in that study were considered.

Finally, questions were asked regarding self-rated health status, financial situation, residence location, and price influence on the quality choice. All measurements were rated on a 7-point anchored scale from 1 (‘strongly disagree’) to 7 (‘strongly agree’). Table 2 presents the items in each scale used in this study.

**Table 2.** Items of the attitudinal measurement scales used in the applied questionnaire.

Attitudinal measurement	Item
Well-Being Scale (Ares et al., 2016)	It is good for well-being
	It makes me feel good
	It is good for my health
	It is nutritious
	It makes me feel satiated
	It helps me control my weight
	It keeps me fit
	It keeps me healthy
	It gives me energy
	It gives me pleasure
	It makes me feel satisfied
	It makes me feel happy
New Naturalness Scale (Michel and Siegrist, 2019)	I make sure to buy products that are preferably free from artificial ingredients.
	I avoid food that contains preservatives.
	I avoid food that contains additives.
	I avoid food that contains artificial colours and flavours.
	I am worried about residues from chemicals in food.
	I avoid food that is made from genetically modified plants.
	It is important to me that foods contain as many natural ingredients as possible.
	I avoid highly processed foods.
	I prefer unprocessed foods over processed foods.
Convenience food lifestyle (Buckley et al., 2007)	We use a lot of ready-to-eat foods in our household
	I choose easy, quick-to-prepare food for weekend evening meals
	I choose easy, quick-to-prepare food for weekday evening meals
	Convenience foods allow me to have something that I wouldn’t normally know how to cook
	I don’t like spending too much time on cooking
	I love spending time in the kitchen preparing food (R)
	Preparing meals gives me a lot of satisfaction (R)

### Data analysis

The participants’ sociodemographic characteristics were summarised using descriptive statistics.

To identify which product attributes and levels most influence the WTT, conjoint analysis was performed to determine the part-worth utilities of the attribute level and the relative importance of attributes, using XL-STAT^®^, v. 2023.1.6 (Addinsoft, New York, NY, USA). The preferred level within each attribute and the most important attributes were identified.

A descriptive statistical analysis was conducted to summarise the sociodemographic variables and determine the mean WTT score for each RTE-rice profile. Friedman's test was employed to evaluate differences in the importance of RTE-rice attributes and assess differences in the WTT between the RTE-rice profiles.

Exploratory factor analysis (EFA) was conducted for each attitude scale using principal component analysis with varimax rotation as the extraction method. Reliability was measured using Cronbach's alpha.

Hierarchical cluster analysis was applied to the factors that emerged from the EFA using the Ward method for agglomeration and square Euclidean distance as the similarity measure. A nonparametric Kruskal–Wallis's test and a pairwise comparison were used to compare the scores for each attitudinal factor between the formed clusters. Differences between the frequency distributions of sex, age, and education level by cluster were evaluated using the chi-square test. All statistical tests were performed at a 95% confidence level. Statistical analyses were performed using the IBM SPSS Statistics version 27.0.

## RESULTS AND DISCUSSION

### Participants’ characterisation

As shown in Table 3, the 106 participants recruited for the study had a mean age of 40 ± 12.8 years old (range, 19–64 years), with equal sex partitions spread across three age groups. Most participants had not completed higher education. Respondents self-reported good health status (5.7 ± 1.23) and financial situation (4.6 ± 1.21). As for sacrificing price for quality or vice versa, they were more inclined to sacrifice price in favour of quality (4.5 ± 1.51). Regarding place of residence, they reported living in a more urban area (2.2 ± 1.76).

**Table 3.** Characterisation of the participants (*n* = 106).

Characteristic	Absolute frequency	Relative frequency (%)
Sex		
Female	54	50.20
Male	52	49.80
Age group (mean ± SD: 40.8 ± 12.8 years)		
[19–34]	34	32.10
[34–49]	40	37.70
[49–64]	32	30.20
Educational level		
No higher education	67	63.2
Higher education	39	36.8
Self-rated health status (mean ± SD)^a^	5.7 (±1.23)	
Self-rated financial situations (mean ± SD)^a^	4.6 (±1.21)	
Trade-off price vs. quality of product^b^	4.5 (±1.51)	
Self-rated urbanisation level (mean ± SD)^c^	2.2 (±1.76)	

a Rating by 7-point scale. It is used to measure self-rated health status from 1 (not at all healthy) to 7 (quite healthy) and financial situation from 1 (difficult) to 7 (quite good).

b How your food expenses are influenced by the cost of products: 1 (price to the detriment of quality) to 7 (quality to the detriment of price).

c Rating by 7-point scale: 1 (urban zone) to 7 (rural zone).

### Conjoint analysis

A recent study on rice consumption habits showed that although Portuguese consumers consider using RTE-rice, they establish a series of criteria, such as sensory aspects, naturalness, and the perception of healthiness (Cabral et al., 2024a). The same study demonstrated that these consumers vary between rice varieties depending on convenience, type of dish, and occasion and that the choice of brown rice is reduced mainly because of the kernel texture. They also demonstrated the importance of information when choosing healthier rice options.

**Figure 3.**
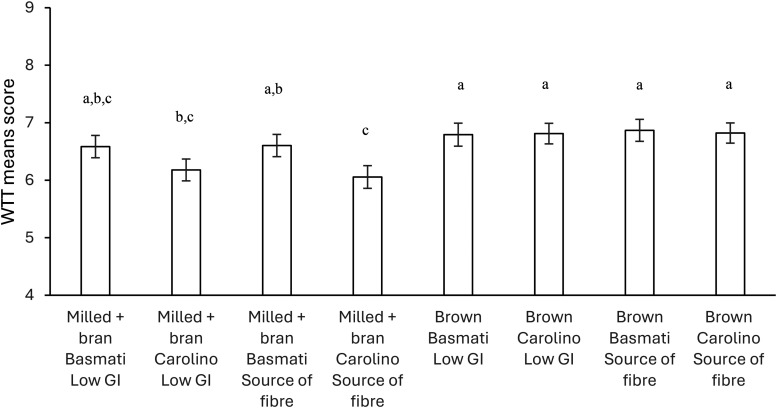
Willingness to try (WTT) means scores (±SE) for each ready-to-eat rice profile.

Consumers scored their WTT RTE-rice as positive, with mean scores varying between 6.1 and 6.9, on a 9-point scale ranging from 1 (‘extremely unwilling’) to 9 (‘extremely willing’). Significantly lower scores in the WTT for RTE-rice made with milled *Carolino* rice with the addition of bran, independently of the claims: ‘Source of fibre’ or ‘Low GI’ (Figure 3).

a, b, c - Significant groups according to the Wilcoxon test, with a significance level of <0.05.

To create eight hypothetical RTE-rice varieties with lower GI, brown rice was used as a base, as it naturally has a lower GI due to the presence of bran, which is commonly removed during milling (Kabir et al., 2021). Adding bran to milled rice improved the nutritional value and GI without changing the texture, a critical factor in rice selection, and the rejection of brown rice (Suwannaporn and Linnemann, 2008). The addition of bran provides nutritional components such as lipids, proteins, dietary fibre, γ-oryzanol, and phytochemical components (Tan et al., 2023), which are associated with a reduced risk of chronic diseases, including cancer (Henderson et al., 2012; Moon and Shibamoto, 2009), lower cholesterol levels (Saji et al., 2019; Zavoshy et al., 2012), and type II diabetes (Cheng et al., 2010; Mahdavi-Roshan et al., 2021). Using bran, typically discarded as waste or used in animal feeds, will reduce waste in the rice processing industry.

Processing and variety attributes had a significantly more significant impact on WTT RTE-rice than claims (Table 4). Based on the part-worth utility value, participants were more WTT RTE-rice with the following attribute levels: brown (0.233), Basmati variety (0.123), and low-GI (0.002). The product comprised of the brown basmati variety and with the low-GI claim showed positive part-worth utilities, indicating that these levels (basmati and brown) increased consumers’ intent to try more than milled Carolino with added bran (Table 4). Claims were less critical, as reported in other joint studies (Carneiro et al., 2005; Rebouças et al., 2021). Although the ‘claim’ attribute showed to be less important (Table 4), it still had an impact on consumer preference, as demonstrated in other studies (Jindahra and Phumpradab, 2023; Rebouças et al., 2019); ‘low-GI’ claims had a more positive effect than ‘Source of fibre’. The two levels of the claim reflect the benefits associated with the product's healthiness; however, they may not have been highly valued because of the food matrices of the products. Studies have found that claims are better accepted for products that are already considered healthy or perceived naturally with the benefits claimed (Ares and Gámbaro, 2007; van Kleef et al., 2005). Therefore, the fibre in the samples would be the most natural; however, the mention of ‘low-GI’ had a more positive effect, contradicting these studies.

**Table 4.** Part-worth utilities and importance for the different attributes of RTE-rice.

Attribute	Level	Part-worth utilities	Mean importance (±SE)	Relative importance (%)
Processing	Brown	0.233	9.7 ± 0.72*	42
	Milled + bran	−0.233		
Variety	Basmati	0.123	8.2 ± 0.64*	36
	*Carolino*	−0.123		
Claim	Low-GI	0.002	4.9 ± 0.47*	22
	Source of fibre	−0.002		

Friedman’s test (two-way analysis of variance by ranks as post hoc), with a 95% confidence level.

* *p* < 0.05.

Brown rice was found to have higher part-worth utilities than milled rice with the addition of bran, despite previous studies showing a high rejection of this type of rice (Cabral et al., 2024a). The same study also confirmed that consumers buy brown rice because it is more nutritious, healthier, and natural than milled rice. Another reason for the preference for brown over milled rice may be the addition of bran, which is an unfamiliar ingredient, as new forms of processing can generate repulsion among consumers, as verified by other studies (Giordano et al., 2018).

Regarding the claims, ‘low-GI’ had higher part-worth utility than nutritional claims related to fibre, despite this already being a claim used in the European market established by Regulation (EC) No. 1924/2006 of the European Parliament and Council. This result contrasts with that of Lähteenmäki (2013), who found that familiarity with a claim positively influences product acceptance. This demonstrates that consumers are interested in and aware of GI’s role in healthier diet choices. Experts in the field have discussed the importance of GI labelling (Augustin et al., 2015; Barclay et al., 2021; Chiu et al., 2011; Wolever, 2013). Although there is no comprehensive regulation for all regions, there seems to be a consensus among competent authorities that low-GI foods and diets generally benefit people with diabetes and impaired glucose metabolism (Yu et al., 2022; Zafar et al., 2019). Food Standards Australia New Zealand provides voluntary guidelines for labelling the GI of food products (GIF, 2022) and has seen significant improvements in GI in the population after its implementation (Yeung et al., 2018). Another example of the application of food GI as an effective tool to control metabolic syndrome is commercial meal-type food for diabetes in South Korea, which the Korean Food and Drug Administration regulates (Lee et al., 2024).

### Consumer attitudes towards rice

The EFA was performed on the psychometric scales administered: the well-being scale, NNS, and CFLs. All scales had a KMO > 0.70 and a significant Bartlett’s Test of Sphericity (<0.001), which demonstrates high correlations between the items that make up each of the factors (Hair et al., 1999).

Table 5 presents the reliability measures of all scale factors with the respective means and standard errors for each item. Cronbach’s alpha revealed very good internal consistency among the factors (Hair et al., 1999).

**Table 5.** Factors resulting from the EFA applied to the items from the different scales (well-being, new naturalness scale, food-related lifestyle).

Factor (Cronbach’s α)	Items	Mean (±SE)
**Emotional **(0.898)		5.5 ± 0.04
	It gives me pleasure	5.4 ± 0.13
	It makes me feel happy	5.2 ± 0.14
	It makes me feel good	5.6 ± 0.11
	It makes me feel satisfied	5.8 ± 0.12
**Physical **(0.882)		5.2 ± 0.04
	It helps me control my weight	4.6 ± 0.15
	It is good for my health	5.5 ± 0.12
	It keeps me healthy	5.4 ± 0.12
	It keeps me fit	4.9 ± 0.14
**General and nutritional **(0.872)		5.6 ± 0.03
	It is nutritious	5.6 ± 0.12
	It gives me energy	5.6 ± 0.12
	It is good for well-being	5.4 ± 0.12
	It makes me feel satiated	5.8 ± 0.10
**New Naturalness Scale **(0.938)		5.5 ± 0.04
	I make sure to buy products that are preferably free from artificial ingredients	5.4 ± 0.16
	I avoid food that contains preservatives	5.3 ± 0.15
	I avoid food that contains additives	5.3 ± 0.16
	I avoid food that contains artificial colours and Flavours	5.7 ± 0.15
	I am worried about residues from chemicals in food	5.8 ± 0.15
	I avoid food that is made from genetically modified plants	4.8 ± 0.18
	It is important to me that foods contain as many natural ingredients as possible	6.0 ± 0.14
	I avoid highly processed foods	5.5 ± 0.15
	I prefer unprocessed foods over processed foods	6.1 ± 0.13
**Convenience food choice** (0.753)		4.0 ± 0.05
	We use a lot of ready-to-eat foods in our household	3.5 ± 0.17
	I choose easy, quick-to-prepare food for weekend evening meals	3.8 ± 0.19
	I choose easy, quick-to-prepare food for weekday evening meals	4.9 ± 0.18
** Convenience in meal preparation and cooking** (0.813)		3.2 ± 0.06
	Convenience foods allow me to have something that I wouldn’t normally know how to cook	4.0 ± 0.18
	I don’t like spending too much time on cooking	3.7 ± 0.20
	I love spending time in the kitchen preparing food	3.3 ± 0.19
	Preparing meals gives me a lot of satisfaction	2.6 ± 0.17

The EFA divided the well-being items into three factors by mixing the original items from the general well-being factor with the physical well-being items. The physical well-being items separated the more nutritional items from the physiological ones, forming the ‘Physical’ and ‘General and nutritional’ factors. The latter includes the item ‘It is good for well-being’, originally belonging to the general factor. The item ‘It makes me feel good’ originally belonged to the general dimension loaded with emotional factors. This item showed a high correlation with the emotional dimension in seven countries in a cross-cultural study on differences in the perception of well-being in the food-related context (Ares et al., 2016).

The factorial structures of the NNS items and CFLs were similar to those reported in the original publications (Buckley et al., 2007; Michel and Siegrist, 2019, respectively). The first CFL factor corresponds to the convenience of food choice items, and the second factor corresponds to the convenience of meal preparation and cooking items, both of which have a very high reliability (Cronbach’s α > 0.75).

Cluster analysis enabled the identification of a pattern of attitudes by applying the six factors originating from EFA. This analysis revealed three distinct groups that exhibited significant differences. The first cluster accorded greater importance to well-being factors and the lowest importance to convenience factors, thereby earning it a well-being-oriented designation. Conversely, the second cluster, which emphasised convenience in meal preparation and cooking factors and had the lowest naturalness importance, was labelled as convenience-oriented. The third cluster attributed greater importance to naturalness; hence, it was labelled as naturalness-oriented. Table 6 lists the characteristics of each cluster.

**Table 6.** Socio-demographic and attitudinal characterisation of the clusters of participants.

Consumer characteristics	Well-being-oriented (*n* = 64)	Convenience-oriented (*n* = 31)	Naturalness-oriented (*n* = 11)	*p*
Sex (%)				ns*
Male	61.5	32.7	5.8	Ns
Female	59.3	25.9	14.8	Ns
Mean age (years) ± SE	43.7 ± 1.58	34.5 ± 1.99	41.4 ± 0.641	<0.001
Age group (years), %				<0.001*
[19–34]	41 (−)	53 (+)	6	
[34–49]	58	28	14	
[49–64]	84 (+)	6 (−)	10	
Education level (%)				<0.01*
Higher education	41 (−)	44 (+)	15	
No higher education	72 (+)	21 (−)	7	
Self-rated health status (mean ± SE)	5.7^b ^± 0.05	5.3^c ^± 0.08	6.4^a ^± 0.05	<0.001
Self-rated financial situation (mean ± SE)	4.5^b ^± 0.06	4.6^b ^± 0.07	5.1^a ^± 0.10	<0.001
Trade-off price vs. quality of product (mean ± SE)	4.6^a ^± 0.07	4.0^b ^± 0.09	4.8^a ^± 0.12	<0.001
Self-rated urbanisation level (mean ± SD)	2.3^a ^± 0.08	2.0^a ^± 0.09	2.5^a ^± 0.24	Ns
Emotional	6.1^a ^± 0.10	4.8^b ^± 0.20	4.2^c ^± 0.27	<0.001
Physical	5.8^a ^± 0.10	4.5^b ^± 0.17	3.8^c ^± 0.30	<0.001
General and nutritional	6.0^a ^± 0.08	5.0^b ^± 0.18	4.6^c ^± 0.31	<0.001
Convenience food choice	3.8^c ^± 0.18	4.1^b ^± 0.20	5.3^a ^± 0.29	<0.01
Convenience in meal preparation and cooking	2.3^c ^± 0.14	4.8^a ^± 0.25	3.8^b ^± 0.37	<0.001
Naturalness	6.1^a ^± 0.09	4.1^b ^± 0.22	6.3^a ^± 0.17	<0.001

a, b - Homogeneous group according to the nonparametric test of Kruskal–Wallis with 5% significance level and the pairwise comparison post hoc test. (+) or (−) indicate that the observed value is significantly higher or lower than the expected value. ns: non-significant.

* Chi-square test per cell with a significance level of 0.05.

Older participants were significantly more inclined towards well-being, whereas younger participants displayed a greater propensity for convenience in prep and cooking. Older consumers are more likely to choose food based on health considerations (Chambers et al., 2008). This corroborates other studies that found that younger consumers did not prepare food from scratch (Mantzari et al., 2020); therefore, they are more open to RTE. The naturalness-orientation group reported significantly better health and financial status, while the convenience-oriented group reported significantly worse health status. Well-being-oriented and naturalness-oriented groups place considerably more importance on quality than price than convenience-oriented consumers.

### Association between product attributes, acceptability, and consumer characteristics

Consumers inclined towards convenience and naturalness were more willing to try RTE-rice than those oriented towards well-being. Naturalness-oriented consumers valued the claimed attribute significantly more and significantly less variety attribute (Table 7).

**Table 7.** Average (±SE) willingness to try, importance and its part-worth utility levels for each attribute according to the cluster grouping based on participants’ attitudes.

Attribute level	Well-being-oriented (*n* = 64)	Convenience-oriented (*n* = 31)	Naturalness-oriented (*n* = 11)	*p*
Willing to try	6.3^b ^± 0.14	6.7^a ^± 0.09	6.7^a ^± 0.17	<0.05
Processing importance	9.1 ± 0.84	10.9 ± 1.51	9.4 ± 2.49	ns
Brown	0.16^b ^± 0.03	0.34^a ^± 0.05	0.36^a ^± 0.05	<0.001
Milled + bran	−0.16^b ^± 0.04	−0.34^a ^± 0.06	−0.36^a ^± 0.06	<0.001
Variety importance	8.8^a ^± 0.80	7.6^b ^± 1.20	6.6^c ^± 2.11	<0.001
Basmati	0.22^a ^± 0.02	−0.04^b ^± 0.04	0.02^a, b ^± 0.02	<0.001
*Carolino*	−0.22^a ^± 0.02	0.04^b ^± 0.04	−0.02^a, b ^± 0.02	<0.001
Claim importance	5.0^b ^± 0.49	4.1^c ^± 1.09	6.9^a ^± 1.80	<0.001
Low-GI	0.03 ± 0.01	−0.04 ± 0.01	−0.02 ± 0.04	ns
Source of fibre	−0.03 ± 0.01	0.04 ± 0.01	0.02 ± 0.04	ns

a, b - Homogeneous group according to the nonparametric test of Kruskal–Wallis with 5% significance level and the pairwise comparison post hoc test. ns: non-significant. The negative signals represent a negative impact on consumer intention to purchase.

The naturalness-oriented group valued convenience in the choice significantly more than the remaining groups and identified with better financial status and a preference for quality at the expense of price. This may indicate that this group perceived the products as natural and was willing to try a convenience product if it met naturalness requirements. This result confirms a change in consumer attitudes towards convenience products in which convenience is no longer directly perceived as unhealthy or unnatural. Researchers have demonstrated that RTE foods can be designed to offer a balanced and healthy meal to support the eating habits of consumers who want or need the advantages of products within this range (Nakano and Washizu, 2020; Stranieri et al., 2017). There is a growing trend towards offering healthier, more sustainable, convenient foods such as naturally preserved fresh vegetables and fruits (Piracci et al., 2024).

Label claims were more important to naturalness-oriented consumers. This attribute, along with other cues, may have reinforced this group's interest in this product category, as they were also the most likely to try it. Label information encourages consumers to appropriately replace their culinary efforts with convenience foods (Nakano and Washizu, 2020).

The group with the lowest WTT RTE-rice was the well-being-oriented group, which attributed less importance to convenience factors and more importance to well-being factors. For this group, it seemed that the fact that it was RTE was more critical in the decision than the ingredients that made up the product. This group was mainly composed of individuals with no higher education, which may have made it difficult to perceive the relationship between the varieties, the process, and the healthiness or benefits of the product. This group was also composed of significantly more people from the older age group, contradicting previous findings that the most likely niche for these products is older (Cabral et al., 2024a). This can be explained by the fact that older consumers are less open to unfamiliar foods (Tuorila et al., 2001) and desire convenience foods that are more similar to homemade foods (Peura-Kapanen et al., 2017), which was not the case for the product profiles evaluated, as the products corresponded to innovative proposals for the participants.

## CONCLUSION

The present study provides initial insights into the perception of carbohydrate-rich foods with GI labelling in Portugal. Although the importance of the GI claim was not a significant factor, it nevertheless positively impacted consumers’ WTT RTE-rice. These findings provide a better understanding of healthy, sustainable, and GI-claimed food products. These insights can inform policies promoting nutritious, sustainable, and convenient staple food consumption. Specifically, the results can help improve consumers’ perceptions of *Carolino* rice, which is essential for the sustainability of the Portuguese rice industry.
